# Editorial: Nanodomain regulation of muscle physiology and alterations in disease

**DOI:** 10.3389/fphys.2022.1092304

**Published:** 2022-11-29

**Authors:** William E Louch, Nina D Ullrich, Manuel F Navedo, Niall Macquaide

**Affiliations:** ^1^ Institute for Experimental Medical Research, Oslo University Hospital, University of Oslo, Oslo, Norway; ^2^ K. G. Jebsen Center for Cardiac Research, University of Oslo, Oslo, Norway; ^3^ Institute of Physiology and Pathophysiology, Division of Cardiovascular Physiology, Heidelberg University, Heidelberg, Germany; ^4^ DZHK (German Center for Cardiovascular Research), Partner Site Heidelberg/Mannheim, Heidelberg University, Heidelberg, Germany; ^5^ Department of Pharmacology, School of Medicine, University of California, Davis, CA, United States; ^6^ School of Health and Life Sciences, Glasgow Caledonian University, Glasgow, United Kingdom

**Keywords:** cardiac, smooth muscle (physiology), excitation-contraction (E-C) coupling, electrophyiology, calcium, phosphorylation, ryanodine receptor (RyR), CaV1.2 Ca channels 2+

## Introduction

Cardiac, skeletal and smooth muscle are vastly different in their function and structure. However, in all three types of muscle, calcium ions (Ca^2+^) serve as the primary second messenger controlling contraction, and this function is modulated by the autonomic nervous system. Disruption of Ca^2+^ signalling and muscle function are evident in pathologies linked to both inherited and acquired conditions, including hypertension, diabetes, muscular dystrophy, and heart disease. Evidence has shown that remodelling of Ca^2+^ signalling nanodomains, as well as other signalling cascades, contribute to these pathological changes. However, our understanding of these mechanisms remains in its infancy. More information is needed to further our comprehension of nanoscale cellular physiology and develop treatments to counteract the changes that occur in disease. This Research Topic has collected 11 high-quality papers from authors focusing on nanodomains and alterations in disease.

## The dyadic space

Early electron microscopy (EM) measurements described the existence of a ∼12–15 nm gap between the sarcolemmal (SL) and sarcoplasmic reticulum (SR) membrane in cardiac and skeletal myocytes called the dyad or triad, respectively (Page and Niedergerke, 1972; Franzini-Armstrong et al., 1999). In healthy ventricular myocytes, most of the estimated ∼20,000 clusters of Ca^2+^ release channels (Ryanodine Receptors, RyRs) are coupled to invaginations of the membrane called transverse(t)-tubules. A sparser network of t-tubules has been observed in atrial myocytes.

## Dyadic remodelling in heart failure

The dyadic space is maintained by junctophilin-2 (JPH2), which spans both SR and SL membranes, and maintains the two membranes in functional dyads. Remodelling of the cardiac dyad has been observed in a host of animal models of heart disease (Heinzel et al., 2002; Quinn et al., 2003; Louch et al., 2004; Song et al., 2006; du Sert et al., 2020) with a loss or remodelling, resulting in an increase in non-coupled (Dries et al., 2013) or orphaned (Song et al., 2006) RyR clusters. Non-coupled RyRs rely on diffusion to trigger Ca^2+^ release resulting in reduced efficiency of Ca^2+^ release. New evidence on nanoscale organisation on JPH2 by Hou et al. shows remodelling of the t-tubule with an increased thickening into branched t-tubule structures called T-sheets, similar to those seen in patient samples observed in block section EM (Pinali et al., 2013), but with no evidence of reduced JPH2 expression. Smaller RyR clusters and reduced RyR expression were also observed, which may contribute to reduced E-C coupling efficiency in heart failure (Beuckelmann et al., 1992; Gómez et al., 2001).

A lack of consensus on correlations of JPH2 expression with t-tubule morphology shows that more work is needed to fully understand the role of this protein in t-tubule sustainment. Indeed, amphiphysin-II (Bin1) is recognised as being an important structural modulator of t-tubule formation and maintenance. Zhou et al. showed the importance of phosphatidylinositol-4,5-bisphosphate (PIP_2_) in this process. Depletion of PIP_2_ caused a loss of t-tubules and a reduction of E-C coupling. New therapies targeting these processes could reverse changes in heart disease.

One observation that does show consistency is the loss of t-tubules in heart failure with reduced ejection fraction. Increasing evidence demonstrates that this subcellular remodelling results in a larger percentage of uncoupled RyRs, with subsequent loss of Ca^2+^ synchrony (Louch et al., 2006). Further ramifications of this form of remodelling are observed through altered regulation of orphaned RyR clusters by kinases and reactive oxygen species (ROS) (Dries et al., 2013; Dries et al., 2018). Indeed, in this issue, Belevych et al. show that detubulated myocytes have a reduced response to sympathetic stimulation upon cholinergic activity. This is the first report of this type of regulation; further work could shed light on a new cholinergic-sympathetic signalling nexus in the ventricular myocyte.

The remodelling of RyR clusters themselves has been reported in cardiac pathologies including heart failure (Kolstad et al., 2018) and atrial fibrillation (Macquaide et al., 2015). Indeed, new imaging information shows altered nanoscale orientation of neighbouring RyRs during acute phosphorylation (Asghari et al., 2020), while chronic phosphorylation during chronic *β*-adrenergic stimulation leads to RyR dispersion (Shen et al., 2022). These observations have ramifications for heart failure, where chronic *β*-adrenergic stimulation and CaMKII activation are well described (Swaminathan et al., 2012). Interestingly, similar remodelling of RyRs was observed in the cerebral microvasculature in Duchenne muscular dystrophy (Pritchard et al., 2018). While Bin1 has been implicated in the recruitment of phosphorylated RyRs to the t-tubules during acute *β*-adrenergic stimulation (Fu et al., 2016), it is unclear whether this protein plays a role in the dispersion of RyRs during longer-term stimulation.

Similar detail is beginning to emerge regarding the plasticity of modular arrangement on the other side of the dyad; i.e., the positioning of L-type Ca^2+^ channels. Clusters of these channels have been reported to increase by 20% in size upon *β*-adrenergic stimulation (del Villar et al., 2021), resulting in increased inter-channel cooperativity in these “superclusters.” These exciting data appear to recapitulate findings from human disease, where increased channel activity and density are observed in human dilated cardiomyopathy patients. This remodelling may be linked to increased CaMKII expression and activity during heart failure (Anderson et al., 2011). Interestingly, Bin1 has also been implicated in the organisation of the L-type Ca^2+^ channel in t-tubules (Hong et al., 2012), although the role of phosphorylation in this process has not been investigated.

## Na^+^ gradients in the dyad

The importance of dyadic Na^+^ ion concentration is highlighted in the review by Skogestad and Aronsen who explore the subcellular localisation and function of the sodium-potassium-ATPase (NKA) in cardiomyocytes. Specifically, they review data indicating that the presence of the alpha-2 isoform (NKAα2) in the dyad vs. NKAα1 outside the dyad may set up subcellular Na^+^ and Ca^2+^ gradients within the myocyte; findings which have implications for the role of NKAα2 in triggering cardiac hypertrophy and arrhythmia. These processes may be important to set the scene for further dyadic remodelling that occurs in disease and may augment the increased NCX activity that is often observed.

## Ageing effects on cAMP microdomains

In the sinoatrial node, pacemaker cells utilise L-type Ca^2+^ signalling as part of the Ca^2+^ clock mechanism, which regulates heart rate. Choi et al. have presented elegant work to correlate nanostructural alterations in aged mice, which leads to declining *β*-adrenergic responsiveness. It appears that fewer *β*-adrenenergic receptors colocalise with L-type Ca^2+^ channels in ageing myocytes, with possible roles for caveolin-3 and AKAP150. This observation highlights that the location of both inside caveolae nanosignalling domains is of utmost importance for their effective interaction.

## New insights from computational modelling and superresolution imaging

In the current Research Topic, the nanoscale structures of ion channel clusters, including RyR and L-type Ca^2+^ channel clusters, are elegantly reviewed by Dixon, with a presented relevance to disease and physiological *β*-adrenergic agonism. Interesting future directions are highlighted, leveraging new super-resolution imaging methods. Indeed, these new imaging technologies have yielded unprecedented levels of structural detail informing present and future modelling endeavours, as reviewed by Louch et al.

Here, new paradigms with high spatial detail are explored with ramifications in disease. The scale of modelling from sub-sarcomere to whole organ is explored in the review by Colman et al. This article highlights the utility and perils of spatial computational modelling in interpreting and scaling up this cellular information to a full organ model with realistic anatomy. The paper by Iaparov et al. is an original model incorporating coupled gating of realistic-sized RyR clusters to explore how Mg^2+^ affects the sensitivity of RyR and how this affects Ca^2+^ release events. This work may provide new information on how drugs requiring Mg^2+^ for their action can be better understood and to produce more realistic models of RyR cluster behaviour.

## Neurohormonal inputs influence muscle nanodomains

A number of our submissions discuss the consequences of sympathetic activity of RyR and L-type Ca^2+^ channel clusters and activity. In Franzoso et al., the role of localised neurotransmitter release from sympathetic nerves is explored in the context of a neuro-cardiac junction akin to the neuro-muscular junction in skeletal muscle. These nerves appear to be very densely distributed, effectively innervating single myocytes, with some myocytes receiving multiple inputs. Ramifications for disease are discussed, introducing interesting new paradigms of localised hyper-adrenergic activation increasing cellular cAMP acting as a trigger for arrhythmic Ca^2+^ release.

Another form of cardiovascular neurohormonal activation is discussed in Salazar-Enciso et al. , where new research shows that upregulation of Ca^2+^ signalling channels and pumps (Ca_v_1.2 and SERCA2) occurs in the plasmalemmal-SR nanodomain in the vascular smooth muscle of mesenteric arteries in response to aldosterone signalling. This increase in SERCA expression importantly occurs in concert with an increased Ca_v_1.2 expression, to modulate Ca^2+^ cycling and prevent vasoconstriction and enhance vasorelaxation.

## Summary

In conclusion, the scientific work presented in this Research Topic has provided intriguing new insight into structure-function relationships within nanosignalling domains. This understanding is expected to provide new avenues for research in cardiovascular disease to facilitate novel, targeted approaches. Indeed, the increasing array of druggable targets offers new hope for tackling complex cardiovascular disease phenotypes. Nevertheless, given the analogies described here, caution must be taken, and an integrative approach should be considered when coordinating the effects of these drugs across multiple organ systems.
